# The Mediating Role of Psychological Inflexibility in the Relationship Between Anxiety, Depression, and Emotional Eating in Adult Individuals With Obesity

**DOI:** 10.3389/fpsyg.2022.861341

**Published:** 2022-04-01

**Authors:** Anna Guerrini Usubini, Giorgia Varallo, Emanuele Maria Giusti, Roberto Cattivelli, Valentina Granese, Simone Consoli, Ilaria Bastoni, Clarissa Volpi, Gianluca Castelnuovo

**Affiliations:** ^1^Istituto Auxologico Italiano IRCCS, Psychology Research Laboratory, Milan, Italy; ^2^Department of Psychology, Catholic University of Milan, Milan, Italy; ^3^Department of Medicine, University of Parma, Parma, Italy; ^4^Department of Psychology, University of Bologna, Bologna, Italy

**Keywords:** anxiety, depression, psychological inflexibility, emotional eating, obesity rehabilitation

## Abstract

The aim of this cross-sectional study is to investigate the role of psychological inflexibility in the relationship between anxiety and depression and emotional eating in a sample of 123 inpatient Italian adult individuals with obesity. Participants completed the Anxiety and Depression subscales of the Psychological General Well-Being Inventory, the Acceptance and Action Questionnaire, and the Emotional Eating subscale of the Dutch Eating Behavior Questionnaire to assess anxiety and depression, psychological inflexibility, and emotional eating, respectively. Results showed that the indirect effect of PGWBI-A on DEBQ-EE through AAQ-II was significant [*b* = −0.0155; SE = 0.076; 95% BC-CI (−0.0320 to −0.023)]. Similarly, the indirect effect of PGWBI-D on DEBQ-EE through AAQ-II was significant [*b* = −0.0383; SE = 0.0207; 95% BC-CI (−0.0810 to −0.0002)]. These findings may help to plan and develop specific psychological interventions aimed at addressing emotional eating through targeting psychological inflexibility to be included in obesity treatment programs.

## Introduction

Obesity is one of the most serious health problems in global public health (Durrer Schutz et al., 2019). Recent estimates indicated that obesity has reached epidemic proportions in the last decades of the 20th century, and its prevalence continues to increase. In 2016 more than 1.9 billion adults in the world were overweight and, of these, more than 650 million were affected by obesity (Castelnuovo et al., 2017).

Defined as excess body fat, obesity is a significant risk factor for a plethora of physical, psychological, and social problems, all of which can heavily impact health, quality of life, and global functioning (Riva et al., 2006; Kolotkin and Andersen, 2017; Afolabi et al., 2020).

In particular, obesity is known to be associated with poor mental health. Depression and anxiety are two of the most common psychological disorders among people with obesity (Preiss et al., 2013; Sharafi et al., 2020), and both depressive and anxiety disorders were found to contribute to weight gain (McNaughton et al., 2008; Strine et al., 2008; Kivimaki et al., 2009). Conversely, social stigma related to obesity can be related to negative body image, low self-esteem, depression, and anxiety, suggesting a bidirectional interplay between obesity, depression, and anxiety (Sahle et al., 2019).

A common risk factor for several disorders including depression and anxiety is psychological inflexibility (Chawla and Ostafin, 2007; Venta et al., 2012). Psychological inflexibility is defined within the model of Acceptance and Commitment Therapy as the “the rigid dominance of psychological reactions over chosen values and contingencies in guiding action” (Hayes et al., 2006; Bond et al., 2011; Strosahl et al., 2012) (Hayes). Psychological inflexibility leads people to avoid negative internal thoughts, feelings, and physical sensations by controlling their behavior, at the expense of more meaningful actions. Psychological inflexibility has been found to be strongly and positively associated with a variety of psychopathologies including anxiety, depression and eating disorders (Rawal et al., 2010). Conversely, it is negatively associated with quality of life, perceived health, and positive emotional experiences (Hayes et al., 2006).

Emotional eating refers to eating in response to unpleasant emotional states (Frayn and Knäuper, 2018). Emotional eating could be problematic for a wide range of physical and psychological problems, as it has been linked to the consumption of unhealthy food and, therefore, weight gain, as well as with poorer psychological wellbeing, depression, and eating disorders (van Strien et al., 1986; Di Renzo et al., 2020; Scarmozzino and Visioli, 2020). Although emotional eating was found both in clinical and non-clinical samples, the prevalence of emotional eating in clinical samples of individuals with obesity is higher, since emotional eating has been associated with elevated consumption of high-calories and high fat (Konttinen et al., 2010). Emotional eating was clearly related to negative emotional states, including depression and anxiety (Whiteside et al., 2007), however underlying factors that explain this relationship are still less studied and should be addressed. Preliminary evidence collected in non-clinical samples suggested that psychological inflexibility and experiential avoidance–the unwillingness to remain in contact and consequently, avoid internal states such as unpleasant thoughts, feelings, sensations–played a key role in the relationship between negative emotional states and the onset of emotional eating (Litwin et al., 2017). Among clinical samples the study of the linkage between negative internal states as anxiety and depression, psychological inflexibility, and emotional eating is increasing (Lillis et al., 2011; Niemeier et al., 2012; Schaumberg et al., 2016; Cattivelli et al., 2021; Guerrini Usubini et al., 2021a,b; Di Sante et al., 2022). Within this perspective, the present study was conceived to replicate and extend literature concerning the role of psychological inflexibility on the relationship between negative internal states, and emotional eating in a clinical sample of inpatient adult individuals with obesity seeking for obesity rehabilitation. As suggested by the literature, we hypothesized to find significant relationships between depression, anxiety, psychological inflexibility, and emotional eating. We also hypothesized that psychological inflexibility may play a role in the linkage between negative internal states of anxiety and depression and emotional eating.

## Materials and Methods

### Participants and Procedures

This cross-sectional study involved 123 Italian adult individuals with obesity, recruited from the Istituto Auxologico Italiano IRCCS, Piancavallo (VB), a specialized clinical center for weight loss and obesity rehabilitation, which is located in the North-West of Italy. Participants were eligible if they met the following inclusion criteria: (1) age between 35 and 65; (2) Body Mass Index (BMI: Kg/m^2^) between 30 and 50 30 (3) Italian mother tongue; and (4) written and informed consent to participate. Participants were excluded if they had any psychiatric disorder diagnosed according to the Diagnostic and Statistical Manual of Mental Disorders (Structured Clinical Interview for DSM-5) criteria including eating disorders, or any concurrent severe medical condition, including neurological and/or physical disabilities, that could compromise the participation in the study.

Participants at the admission to the hospital were informed about their participation in the study and were selected for recruitment with a clinical interview conducted by a clinical psychologist. Once enrolled, participants were asked to complete an anonymous survey *via* self-report form. Data were collected from 1st December 2019 to 3rd March 2020. The Medical Ethics Committee of Istituto Auxologico Italiano approved the study and all procedures on human subjects were conducted following the Helsinki Declaration of 1975, as revised in 1983.

### Measures

Demographic (sex, age, educational level, marital status, and work status), physical (weight in kg and height in m to calculate BMI with the following formula: BMI = kg/m^2^) and clinical data were collected through a self-report form. Clinical variables were collected as follows:

#### Anxiety and Depression

To assess anxiety and depression, we used the Anxiety and Depression subscales of the Psychological General Well-Being Inventory (PGWBI-A and PGWBI-D) (Dupuy, 1990). The PGWBI is a well-known and validated measure that provides a general subjective assessment of psychological wellbeing and health. The whole questionnaire comprises 22 self-administered items rated on a 6-point Likert scale, exploring six dimensions: anxiety, depression, positive wellbeing, self-control, general health, and vitality. The Anxiety subscale is composed of five items while the Depression subscale is composed of three items. Higher scores in Anxiety and Depression subscales indicate less anxiety and depression, while lower scores in those subscales suggest greater suffering. We used the Italian version (Grossi et al., 2002) that showed good psychometric properties (Cronbach’s alpha of Anxiety subscale of the Italian validation = 0.85; Cronbach’s alpha of Depression subscale of the Italian validation = 0.80; Rossi et al., 2021), in agreement with the original version.

#### Psychological Inflexibility

To assess psychological inflexibility, we used the Acceptance and Action Questionnaire (AAQ-II) (Bond et al., 2011) that comprises 10 items rated on a 7-point Likert scale. It is the most widely used self-reported questionnaire that provides a measure of psychological inflexibility. Higher scores indicated higher inflexibility. We used the Italian version (Berrocal et al., 2013) that showed good psychometric properties (Cronbach’s alpha = 0.77), in agreement with the original version.

#### Emotional Eating

To assess emotional eating, we used the Emotional Eating subscale of the Dutch Eating Behavior Questionnaire (DEBQ-EE) (van Strien et al., 1986). It is composed of 13 of the 33 total items of the DEBQ rated on a 5-point scale (1 = never, 5 = very often). Higher scores indicated higher emotional eating. We used the Italian version (Dakanalis et al., 2013) that showed good psychometric properties (Cronbach’s alpha of Emotional eating subscale in the subsample of overweight people = 0.97), in agreement with the original version.

### Statistical Analysis

Descriptive statistics were calculated using means and standard deviation for continuous variables, and frequencies and percentages for categorical variables. To assess normal distribution of variables, skewness and kurtosis were evaluated. Parameters outside the limit of +2/−2 range were considered an index of non-normality (Podsakoff et al., 2003). Missing data were lower than 5%, and therefore were considered inconsequential (Schafer et al., 2016). In all analyses, statistical significance was set to *p* < 0.05.

Bivariate Pearson’s correlations were used to assess correlations between all continuous demographical (age), physical (BMI), and clinical variables (PGWBI-A, PGWBI-D, AAQ-II, and DEBQ-EE). Independent sample *t*-test performed to assess differences in emotional eating (DEBQ-EE) between males and females.

To investigate the role of psychological inflexibility between negative internal states and emotional eating, two mediation models were tested with PGWBI-A and PGWBI-D included as predictors respectively, AAQ-II as mediator, and DEBQ-EE as outcome. Sex was entered as covariate in both models. Mediations were run using Model 4 of PROCESS Macro for SPSS. An estimation of the indirect effect was obtained using Maximum Likelihood estimator. Bias-corrected bootstrapping method (5,000 samples) was used to calculate 95% bias-corrected confidence intervals (BC-CIs) to determine the significance of the mean indirect effects. The indirect effect was considered statistically significant at *p* < 0.05 when 95% BC-CIs did not include zero (Preacher and Hayes, 2008). According to Fritz and Mackinnon’s empirical power tables for mediation models, a sample size of 115 is sufficient to find a mediated effect including small-to-medium (0.26) effect of β with α of 0.05 (Fritz and MacKinnon, 2007). We performed analyses using IBM Statistical Package for the Social Sciences SPSS version 26 (Armonk, NY, United States: IBM Corp).

## Results

### Descriptive Statistics of the Sample and Relations Among Variables

The sample was composed of 69 (56.1%) females and 54 males (43,9%) aged between 35 and 65 (*M* = 53.7; *SD* = 6.98), the average BMI was 41.4 (*SD* = 3.70). Descriptive statistics of the sample and measured variables characteristics were presented in Supplementary Table 1.

Pearson’s bivariate correlations showed that there was a significant correlation between PGWBI-A and DEBQ-EE, (*r* = −0.384; *p* < 0.001) as well as between PGWBI-D and DEBQ-EE (*r* = −0.292; *p* < 0.001). The AAQ-II was significantly and positively related to DEBQ-EE (*r* = 0.346; *p* < 0.001). The independent sample *t*-test revealed that females (*M* = 3.18; 1.19) reported higher levels of DEBQ-EE than males (*M* = 2.79; *SD* = 0.85) (*t* = 2.10; *p* = 0.038) Results are presented in Supplementary Table 2.

### Mediation Model

Results showed that the indirect effect of of PGWBI-A on DEBQ-EE through AAQ-II was significant [*b* = −0.0155; SE = 0.076; 95% BC-CI (−0.0320 to −0.023)]. In addition, the direct effect PGWBI-A on DEBQ-EE was significant [*b* = −0.0546; SE = 0.0179; *p* = 0.0282; 95% BC-CI (−0.0900 to −0.0192)]. Finally, the total effect of PGWBI-A on DEBQ-EE was significant [*b* = −0.0701; SE = 0.0168; *p* < 0.001; 95% BC-CI (−0.1033 to 0.0369)]. The results also suggest that the indirect mediated effect accounted for 19% (*R*^2^ = 0.1993) of the variance.

Similarly, the indirect effect of PGWBI-D on DEBQ-EE through AAQ-II was significant [*b* = −0.0383; SE = 0.0207; 95% BC-CI (−0.0810 to −0.0002)]. In addition, the direct effect of PGWBI-D on DEBQ-EE was not significant [*b* = −0.07301; SE = 0.0417; *p* = 0.0823; 95% BC-CI (−0.1556–0.0095)]. Finally, the total effect of PGWBI-D on DEBQ-EE was significant [*b* = −0.1113; SE = 0.0370; *p* = 0.032; 95% BC-CI (−0.1846 to −0.0381)]. The results also suggest that the indirect mediated effect accounted for 12% (*R*^2^ = 0.1284) of the variance.

**FIGURE 1:**
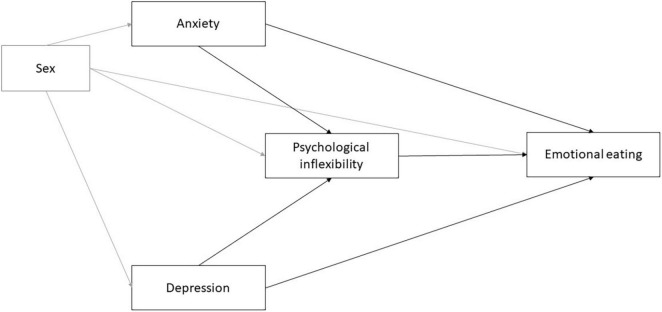
The hypothesized models.

Path models are presented in Figure 1.

## Discussion

The current study examined the relationship between anxiety and depression, psychological inflexibility, and emotional eating in a sample of adult individuals with obesity, exploring psychological inflexibility as part of a potential mechanism linking negative internal states and eating pathology.

In line with our hypothesis, we found significant associations between anxiety and emotional eating and between depression and emotional eating (Ouwens et al., 2009; Guerrini Usubini et al., 2021a), in which higher levels of anxiety and depression were related to higher emotional eating. Correlations between anxiety and depression and psychological inflexibility were significant, suggesting that the higher the level of anxiety and depression, the higher the level of psychological inflexibility. These results were consistent with previous findings pointing out that psychological inflexibility was positively related to a range of psychological problems, including mood disturbances and anxiety disorders (Venta et al., 2012).

We also found that psychological inflexibility played a significant indirect effect in the association between depression and emotional eating, and also in the association between anxiety and emotional eating.

These results are in line with previous findings exploring possible pathways between depression anxiety and dysfunctional eating behaviors (i.e., emotional and external eating) suggesting that psychological inflexibility was associated with both depression and anxiety in a sample of female participants (Ouwens et al., 2009). In addition, another study by Litwin et al. (2017) found that, among undergraduated female students, experiential avoidance was a significant mediator of the relationship between negative emotions (measured with the Positive and negative affect scheduled) and emotional eating. In both studies, however, the sample consisted exclusively of female participants. In contrast, our study includes both female and male participants diagnosed with obesity, allowing the results to be extended to this clinical population.

Similar findings are also consistent with the affect regulation model positing that negative emotions are predictors of emotional eating (Spoor et al., 2007; Goossens et al., 2009). Thus, emotional eating might serve to reduce the intensity of negative emotions (Spoor et al., 2007; Haedt-Matt and Keel, 2011; Macht and Simons, 2011; Haedt-Matt et al., 2014; Litwin et al., 2017; Guerrini Usubini et al., 2021).

Furthermore these results are in live also with the conceptual model of Forman and Butryn (2015) which suggested that long-standing adherence to a healthy lifestyle was associated with some self-regulation skills, such as distress tolerance and values clarity which can help individuals cope with internal and external stimuli coherently with personal values and goals (Kashdan and Rottenberg, 2010; Varallo et al., 2021). The self-regulation skills have been found to play a protective role against overreacting to internal (e.g., emotions) and external (e.g., the availability of palatable food in the environment) cues that motivate people to eat palatable food, as in the case of emotional eating. On the contrary, psychological inflexibility leads people to avoid negative internal thoughts, feelings, and physical sensations by controlling engaging in maladaptive behaviors such as emotional eating, at the expense of more meaningful actions (Levin et al., 2013).

Our findings also suggest that emotional eating may be used as a strategy to change or avoid unpleasant internal experiences, indicating that emotional eating is determined not only by the presence of negative emotions, but also by the subject’s response to negative emotions (i.e., avoidance). Therefore, a reduction of psychological inflexibility could also contribute to a reduction of emotional eating by promoting a greater willingness to accept negative emotions without trying to avoid them.

Our results can be seen in light of their clinical implications. Because psychological inflexibility appears to play a role in both the anxiety-emotional-eating relationship and the depression-emotional-eating relationship, interventions aimed at promoting psychological flexibility could have a significant impact on how both anxiety and depression are managed. This hypothesis should be tested in future research. In addition, our results suggest that interventions specifically aimed at reducing psychological inflexibility, such as Acceptance and commitment therapy (ACT) or dialectical behavior therapy (DBT) may be effective for reducing emotional eating. Indeed, ACT and DBT have been shown to be effective in the treatment of eating disorders and weight issues (Forman et al., 2009; Juarascio et al., 2013).

To sum up, the current study adds to our understanding of emotional eating. The findings presented fill a critical gap in the literature by implying that negative emotions are linked to increased levels of emotional eating *via* psychological inflexibility. However, psychological inflexibility has a partial indirect effect in the relationship between negative emotions and emotional eating, implying that other factors, such as for example self-efficacy or emotion dysregulation, should be investigated in future research. Although this investigation adds knowledge on emotional eating in obesity, several limitations of the study must be discussed. This study used a cross-sectional design, and so, the nature of the current study warrants cautions to do causality conclusions. To overcome this limitation, future studies should be planned with the inclusion of comparison with a normal-weight group and longitudinal measurements. Furthermore, in this study, we used only self-report measures that could be affected by bias and limitations (e.g., social desirability). Direct, instead of retrospective measures could be free from biases, such as one’s current emotional state, and provide an objectively assess the intended construct. Finally, quite small relationships were found, suggesting that there could be additional factors that we did not consider in our study that need to be addressed in future research replications. Another limitation concerns the sample representativeness. The findings represent a sample of Italian hospitalized individuals seeking treatment for obesity and may not necessarily reflect individuals in other conditions. Future replications of the study should also involve different samples. Finally, future replications of the study are needed to explore the role of additional variables, including alexithymia and interoceptive awareness, which were related to emotional eating (McDowell et al., 2002; Van Strien et al., 2005; van Strien, 2018). In addition, it may be useful to explore these relationships in clinical samples of people with obesity and comorbid eating disorders (e.g., binge eating disorder).

Despite limitations, our study provides important strengths and clinical implications. By elucidating the role of psychological inflexibility between negative affect and emotional eating, our results deepen the understanding of mechanisms behind dysfunctional eating patterns, by providing specific evidence in a clinical sample of individuals with obesity.

## Conclusion

Our results suggest that psychological inflexibility has an indirect effect in the relationship between negative states of anxiety and depression and emotional eating in Italian adult individuals with obesity. Our findings suggest that developing specific psychological interventions for overweight and obesity that target the process of psychological inflexibility might be beneficial for reducing emotional eating.

## Data Availability Statement

The raw data supporting the conclusions of this article will be made available by the authors, without undue reservation.

## Ethics Statement

The studies involving human participants were reviewed and approved by the Istituto Auxologico Italiano. The patients/participants provided their written informed consent to participate in this study.

## Author Contributions

AG and GV conceived the study, planned the design, made a substantial contribution to the manuscript drafting, defined the statistical analysis, and establish the sample size for the study. VG, RC, SC, IB, and CV contributed greatly to the manuscript drafting. GC and EG read and approved the final manuscript. All authors contributed to the article and approved the submitted version.

## Conflict of Interest

The authors declare that the research was conducted in the absence of any commercial or financial relationships that could be construed as a potential conflict of interest.

## Publisher’s Note

All claims expressed in this article are solely those of the authors and do not necessarily represent those of their affiliated organizations, or those of the publisher, the editors and the reviewers. Any product that may be evaluated in this article, or claim that may be made by its manufacturer, is not guaranteed or endorsed by the publisher.
