# Corrigendum: The mediating role of psychological inflexibility in the relationship between anxiety, depression, and emotional eating in adult individuals with obesity

**DOI:** 10.3389/fpsyg.2022.1035132

**Published:** 2022-10-26

**Authors:** Anna Guerrini Usubini, Giorgia Varallo, Emanuele Maria Giusti, Roberto Cattivelli, Valentina Granese, Simone Consoli, Ilaria Bastoni, Clarissa Volpi, Gianluca Castelnuovo

**Affiliations:** ^1^Istituto Auxologico Italiano IRCCS, Psychology Research Laboratory, Milan, Italy; ^2^Department of Psychology, Catholic University of Milan, Milan, Italy; ^3^Department of Medicine, University of Parma, Parma, Italy; ^4^Department of Psychology, University of Bologna, Bologna, Italy

**Keywords:** anxiety, depression, psychological inflexibility, emotional eating, obesity rehabilitation

In the published article, the **Funding** statement was missing. The correct **Funding** statement appears below.

## Funding

This study was funded by the Italian Ministry of Health.

The authors apologize for this error and state that this does not change the scientific conclusions of the article in any way. The original article has been updated.

## Publisher's note

All claims expressed in this article are solely those of the authors and do not necessarily represent those of their affiliated organizations, or those of the publisher, the editors and the reviewers. Any product that may be evaluated in this article, or claim that may be made by its manufacturer, is not guaranteed or endorsed by the publisher.

