# Supplementary material for: The Mediating Role of Psychological Inflexibility in the Relationship Between Anxiety, Depression, and Emotional Eating in Adult Individuals With Obesity
Source: Front Psychol. 2022 Apr 1;13:861341. doi: 10.3389/fpsyg.2022.861341 (PMC9012297; doi:10.3389/fpsyg.2022.861341)
Supplement: Supplementary file 1 [file Table_1.DOCX]

**Supplementary materials**

Table 1. Descriptive statistics of the sample

|  | | | |
| --- | --- | --- | --- |
|  | N (%) | Mean ± SD | Range |
| Sex |  |  |  |
| Male | 77(45.3) |  |  |
| Female | 93 (54.7) |  |  |
| Age (in years) | 170 | 51.1± (10.4) | 18-65 |
| BMI (Kg/m^2^) | 166 | 44.2 ± (8.28) |  |
| Educational level |  |  |  |
| Primary school | 8 (4.7%) |  |  |
| Secondary school | 46 (27.1%) |  |  |
| Higher school | 81 (47.6%) |  |  |
| Bachelor's degree | 7 (4.1%) |  |  |
| Master's degree | 26 (15.3%) |  |  |
| Marital Status |  |  |  |
| Single | 62 (36.5%) |  |  |
| Married | 79 (46.5%) |  |  |
| Divorced | 20 (11.8%) |  |  |
| Widowed | 8 (4.7%) |  |  |
| Work status |  |  |  |
| Student | 78 (45.9%) |  |  |
| Employed | 24 (14.1%) |  |  |
| Unemployed | 5 (2.9%) |  |  |
| Housewife | 33 (19.4%) |  |  |
| Retired | 30 (17.6%) |  |  |
| PGWBI-A | 169 | 15.6 ± (5.41) | 1-25 |
| PGWBI-D | 169 | 11.5 ± (2.98) | 1-15 |
| AAQ-II | 166 | 34.6 ± (11.8) | 10-61 |
| DEBQ-EE | 166 | 2.98 ± (1.08) | 1-5 |
| Note: BMI: Body Mass Index; PGWBI_A: Psychological General Well-being Index_Anxiety subscale; PGWBI_D: Psychological General Well-being Index_Depression subscale; DEBQ- EE: Dutch Eating Behavior Questionnaire_Emotional Eating subscale; AAQ-II: Acceptance and Action Questionnaire-II; | | | |

| Table 2. Relations among variables | | | | | | | |  |
| --- | --- | --- | --- | --- | --- | --- | --- | --- |
|  | Age | BMI | PGWBI-A | PGWBI_D | AAQ-II | DEBQ_EE | F | p |
| Age | - |  |  |  |  |  |  |  |
| BMI | -0.097 | - |  |  |  |  |  |  |
| PGWBI-A | 0.113 | -0.036 | - |  |  |  |  |  |
| PGWBI-D | 0.082 | -0.026 | 0.782*** | - |  |  |  |  |
| AAQ-II | -0.215 | 0.065 | -0.422*** | -0.514*** | - |  |  |  |
| DEBQ-EE | -0.230 | -0.004 | -0.397*** | -0.376*** | 0.406*** | - |  |  |
| sex |  |  |  |  |  |  | 6.22 | .014 |
| Note:PGWBI_A: Psychological General Well-being Index_Anxiety subscale; PGWBI_D: Psychological General Well-being Index_Depression subscale; DEBQ- EE: Dutch Eating Behavior Questionnaire_Emotional Eating subscale; AAQ-IIAcceptance and Action Questionnaire-II;  *** p < .001 | | | | | | | |  |
